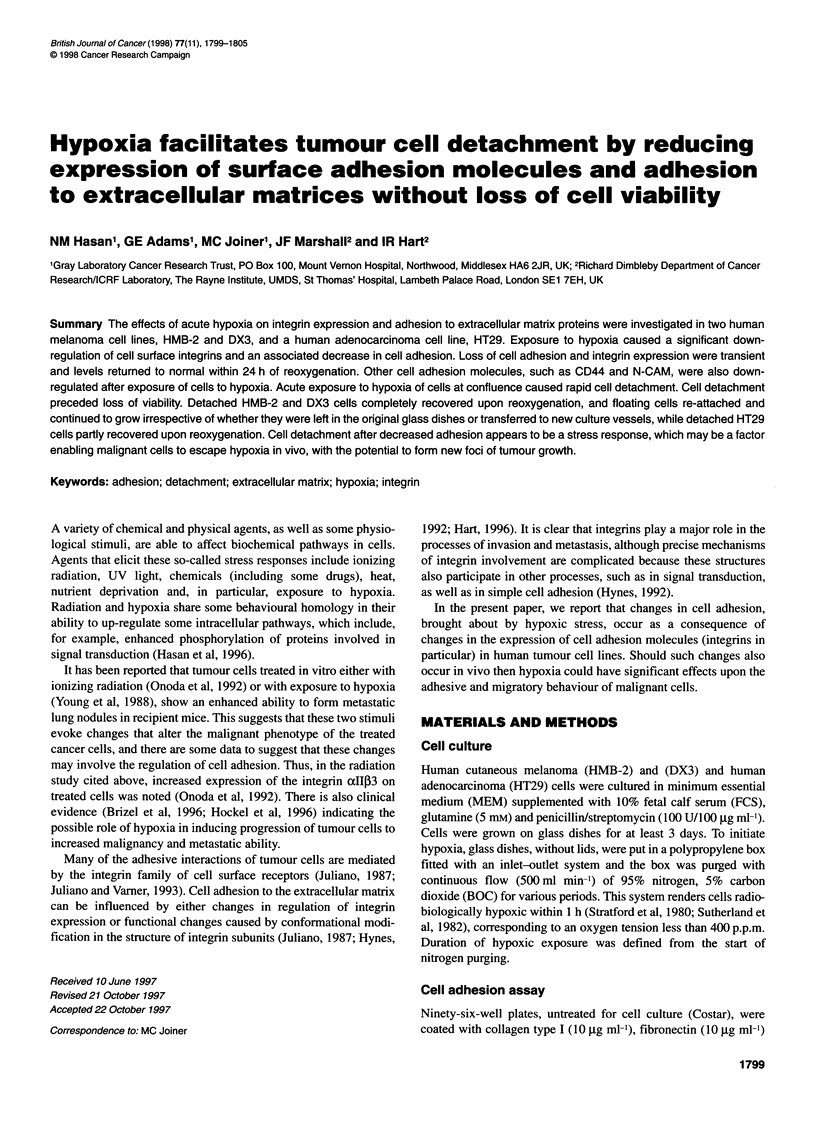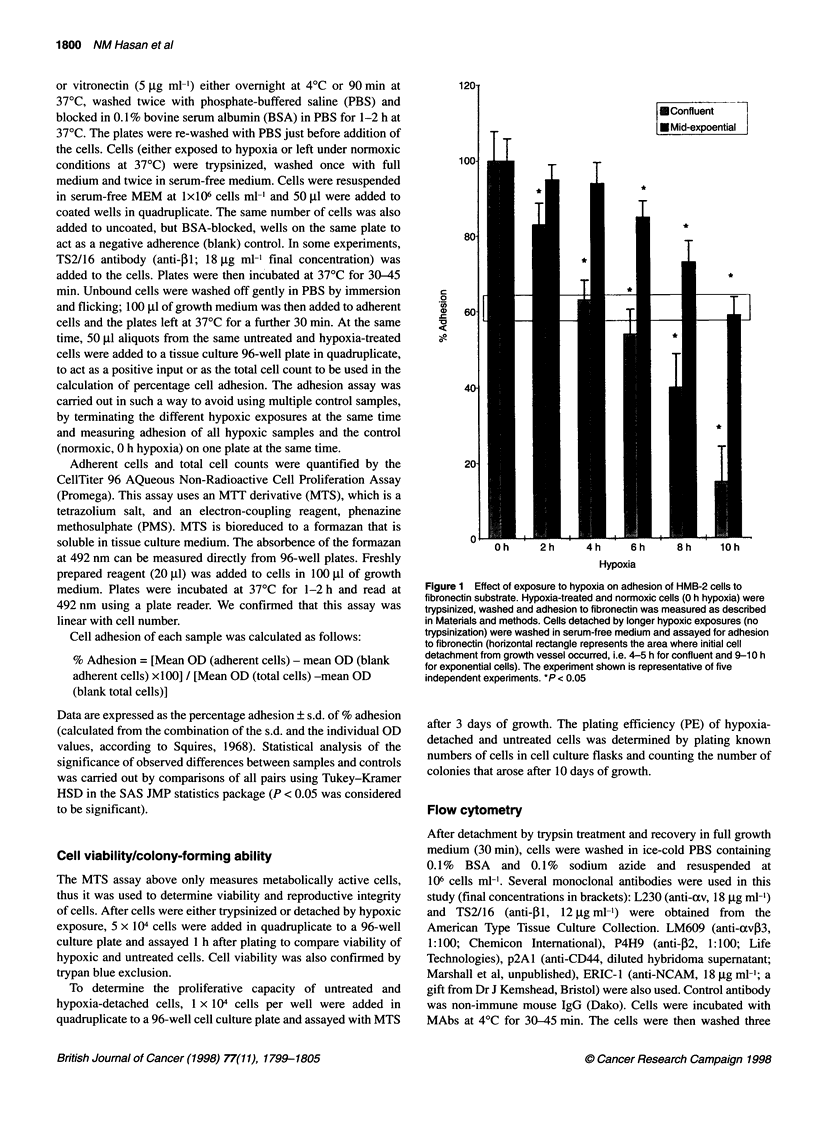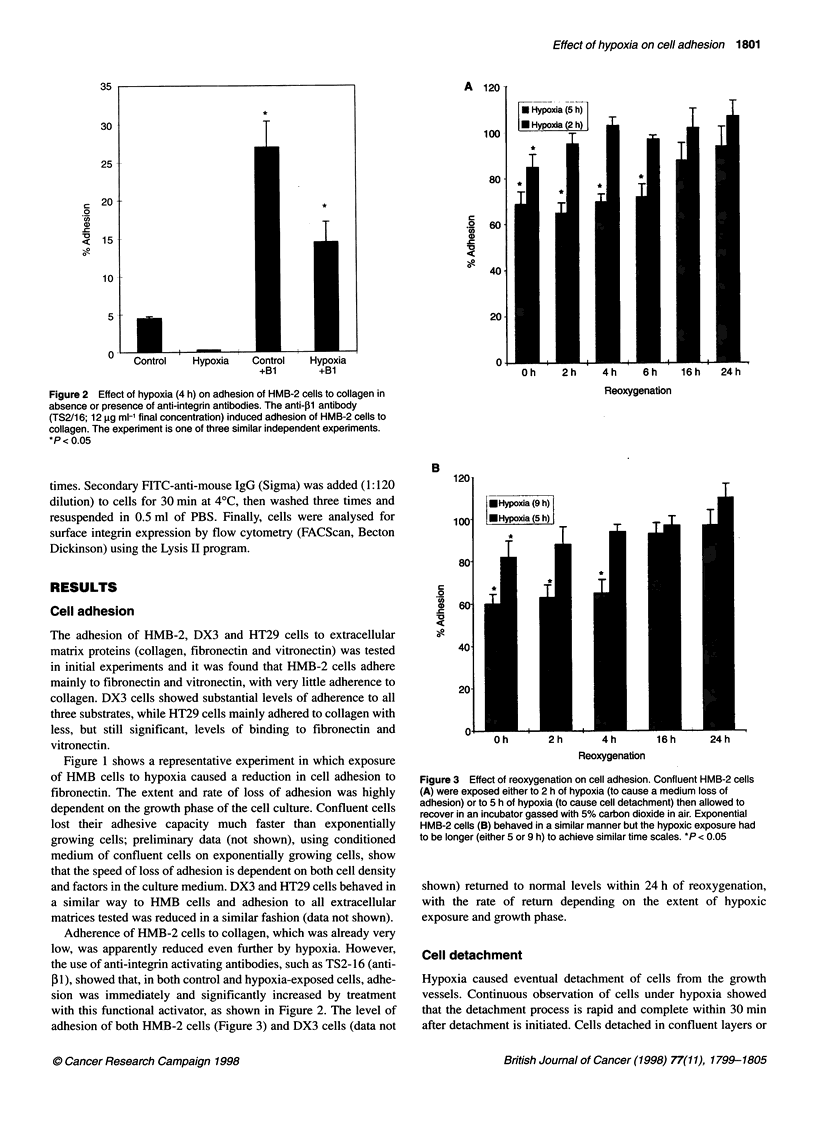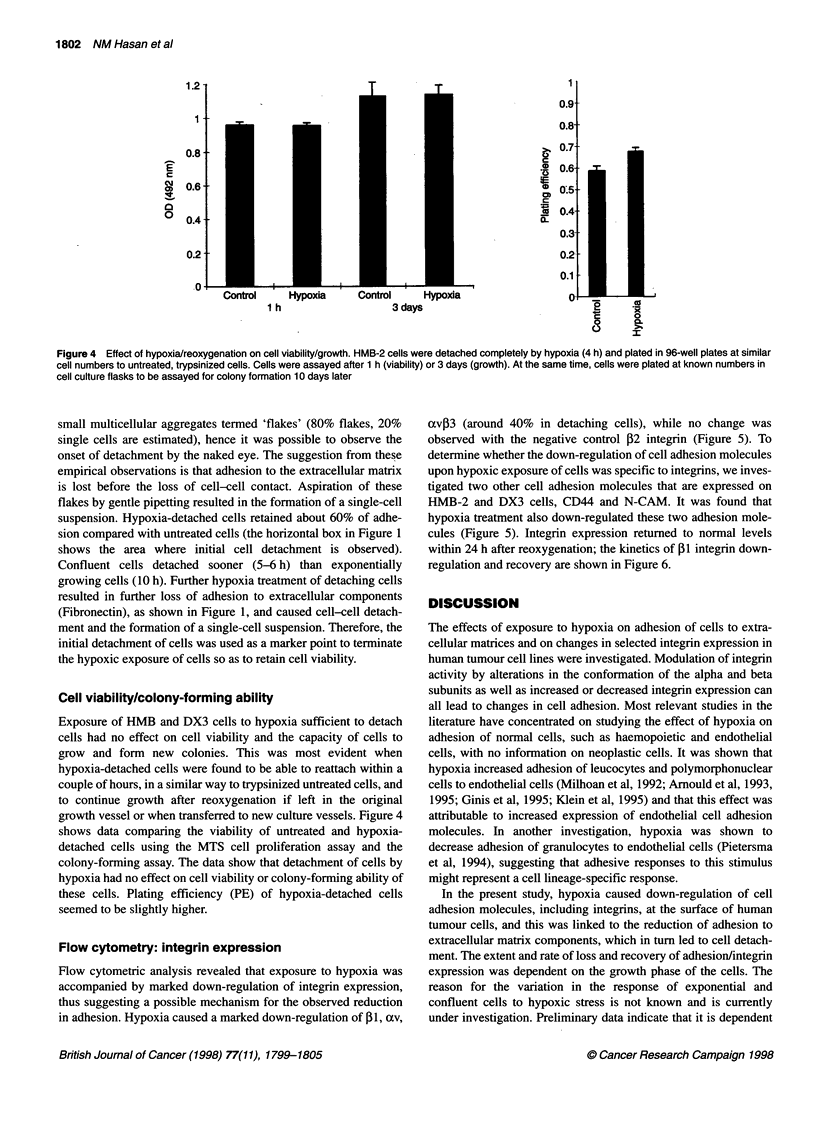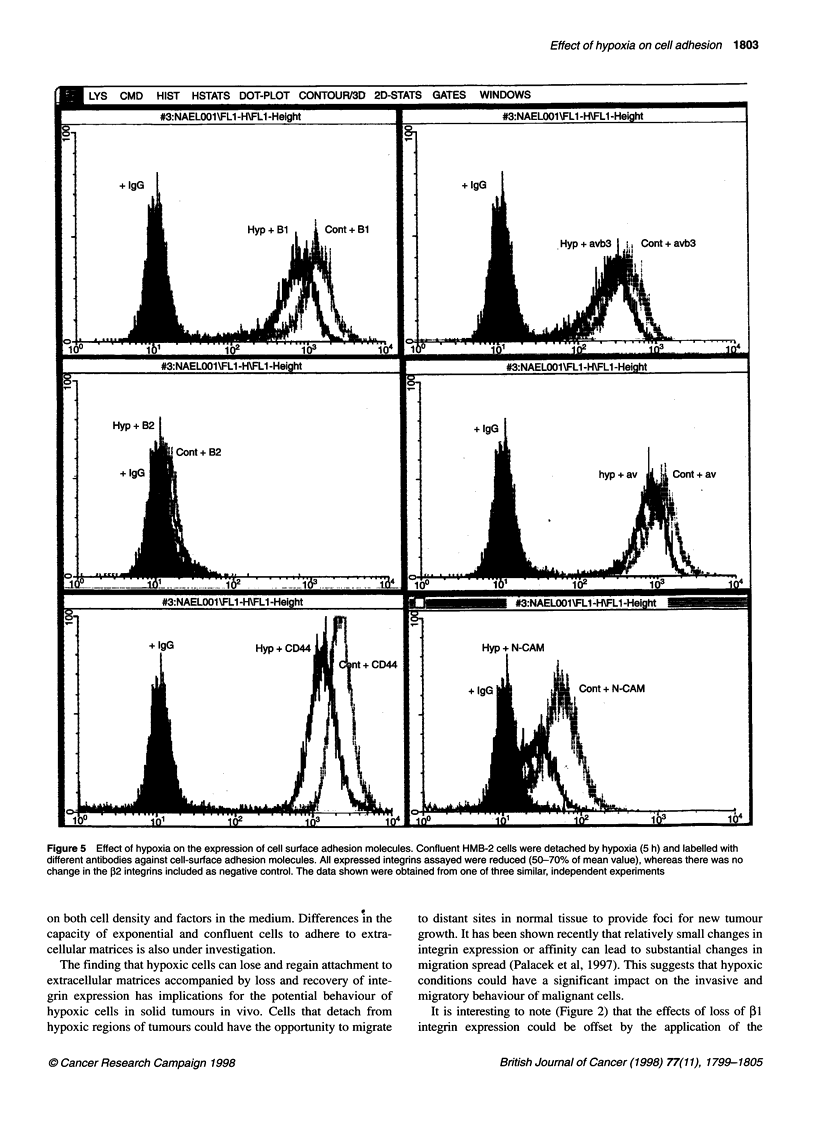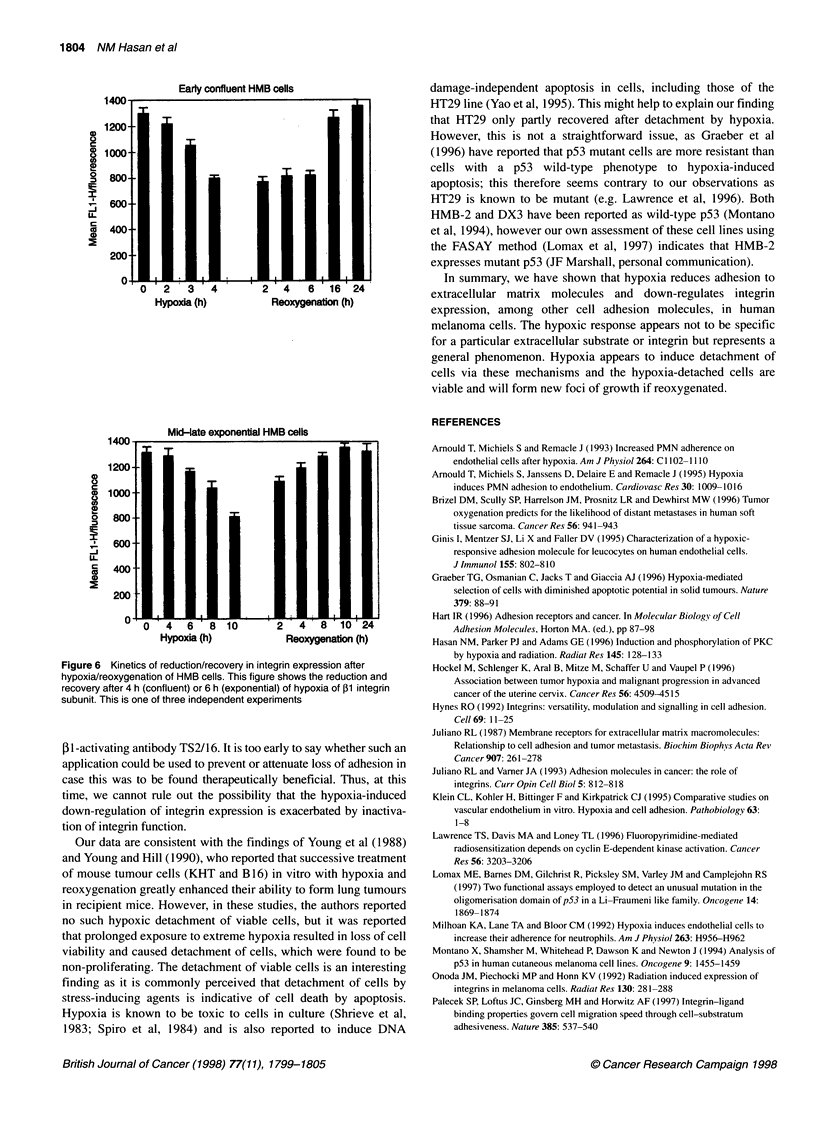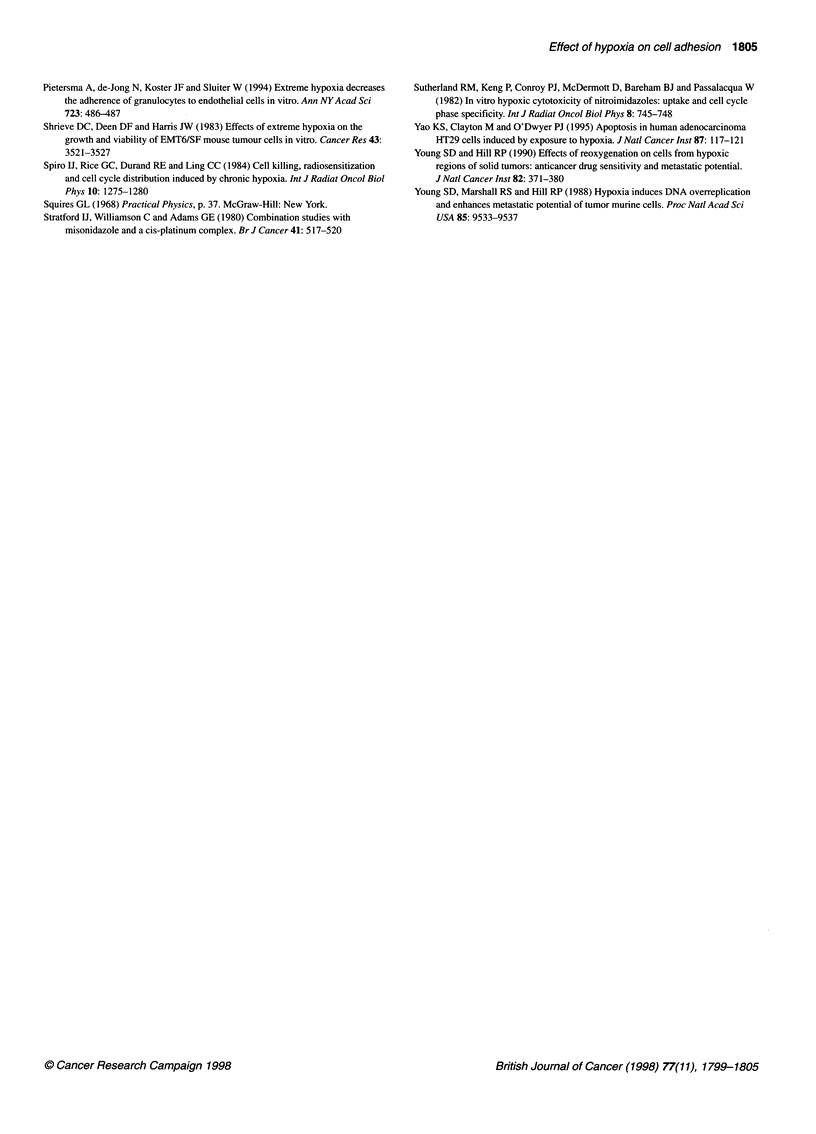# Hypoxia facilitates tumour cell detachment by reducing expression of surface adhesion molecules and adhesion to extracellular matrices without loss of cell viability.

**DOI:** 10.1038/bjc.1998.299

**Published:** 1998-06

**Authors:** N. M. Hasan, G. E. Adams, M. C. Joiner, J. F. Marshall, I. R. Hart

**Affiliations:** Gray Laboratory Cancer Research Trust, Mount Vernon Hospital, Northwood, Middlesex, UK.

## Abstract

The effects of acute hypoxia on integrin expression and adhesion to extracellular matrix proteins were investigated in two human melanoma cell lines, HMB-2 and DX3, and a human adenocarcinoma cell line, HT29. Exposure to hypoxia caused a significant down-regulation of cell surface integrins and an associated decrease in cell adhesion. Loss of cell adhesion and integrin expression were transient and levels returned to normal within 24 h of reoxygenation. Other cell adhesion molecules, such as CD44 and N-CAM, were also down-regulated after exposure of cells to hypoxia. Acute exposure to hypoxia of cells at confluence caused rapid cell detachment. Cell detachment preceded loss of viability. Detached HMB-2 and DX3 cells completely recovered upon reoxygenation, and floating cells re-attached and continued to grow irrespective of whether they were left in the original glass dishes or transferred to new culture vessels, while detached HT29 cells partly recovered upon reoxygenation. Cell detachment after decreased adhesion appears to be a stress response, which may be a factor enabling malignant cells to escape hypoxia in vivo, with the potential to form new foci of tumour growth.


British Journal of Cancer (1998) 77(11), 1799–1805
© 1998 Cancer Research Campaign

### Hypoxia facilitates tumour cell detachment by reducing expression of surface adhesion molecules and adhesion to extracellular matrices without loss of cell viability

**NM Hasan[1], GE Adams[1], MC Joiner[1], JF Marshall[2] and IR Hart[2]**

[1]Gray Laboratory Cancer Research Trust, PO Box 100, Mount Vernon Hospital, Northwood, Middlesex HA6 2JR, UK; [2]Richard Dimbleby Department of Cancer Research/ICRF Laboratory, The Rayne Institute, UMDS, St Thomas' Hospital, Lambeth Palace Road, London SE1 7EH, UK

**Summary** The effects of acute hypoxia on integrin expression and adhesion to extracellular matrix proteins were investigated in two human melanoma cell lines, HMB-2 and DX3, and a human adenocarcinoma cell line, HT29. Exposure to hypoxia caused a significant down-regulation of cell surface integrins and an associated decrease in cell adhesion. Loss of cell adhesion and integrin expression were transient and levels returned to normal within 24 h of reoxygenation. Other cell adhesion molecules, such as CD44 and N-CAM, were also down-regulated after exposure of cells to hypoxia. Acute exposure to hypoxia of cells at confluence caused rapid cell detachment. Cell detachment preceded loss of viability. Detached HMB-2 and DX3 cells completely recovered upon reoxygenation, and floating cells re-attached and continued to grow irrespective of whether they were left in the original glass dishes or transferred to new culture vessels, while detached HT29 cells partly recovered upon reoxygenation. Cell detachment after decreased adhesion appears to be a stress response, which may be a factor enabling malignant cells to escape hypoxia in vivo, with the potential to form new foci of tumour growth.

**Keywords:** adhesion; detachment; extracellular matrix; hypoxia; integrin

A variety of chemical and physical agents, as well as some physiological stimuli, are able to affect biochemical pathways in cells. Agents that elicit these so-called stress responses include ionizing radiation, UV light, chemicals (including some drugs), heat, nutrient deprivation and, in particular, exposure to hypoxia. Radiation and hypoxia share some behavioural homology in their ability to up-regulate some intracellular pathways, which include, for example, enhanced phosphorylation of proteins involved in signal transduction (Hasan et al, 1996).

It has been reported that tumour cells treated in vitro either with ionizing radiation (Onoda et al, 1992) or with exposure to hypoxia (Young et al, 1988), show an enhanced ability to form metastatic lung nodules in recipient mice. This suggests that these two stimuli evoke changes that alter the malignant phenotype of the treated cancer cells, and there are some data to suggest that these changes may involve the regulation of cell adhesion. Thus, in the radiation study cited above, increased expression of the integrin $\alpha II\beta 3$ on treated cells was noted (Onoda et al, 1992). There is also clinical evidence (Brizel et al, 1996; Hockel et al, 1996) indicating the possible role of hypoxia in inducing progression of tumour cells to increased malignancy and metastatic ability.

Many of the adhesive interactions of tumour cells are mediated by the integrin family of cell surface receptors (Juliano, 1987; Juliano and Varner, 1993). Cell adhesion to the extracellular matrix can be influenced by either changes in regulation of integrin expression or functional changes caused by conformational modification in the structure of integrin subunits (Juliano, 1987; Hynes, 1992; Hart, 1996). It is clear that integrins play a major role in the processes of invasion and metastasis, although precise mechanisms of integrin involvement are complicated because these structures also participate in other processes, such as in signal transduction, as well as in simple cell adhesion (Hynes, 1992).

In the present paper, we report that changes in cell adhesion, brought about by hypoxic stress, occur as a consequence of changes in the expression of cell adhesion molecules (integrins in particular) in human tumour cell lines. Should such changes also occur in vivo then hypoxia could have significant effects upon the adhesive and migratory behaviour of malignant cells.

*Received 10 June 1997*
*Revised 21 October 1997*
*Accepted 22 October 1997*

*Correspondence to:* MC Joiner

### MATERIALS AND METHODS

### Cell culture

Human cutaneous melanoma (HMB-2) and (DX3) and human adenocarcinoma (HT29) cells were cultured in minimum essential medium (MEM) supplemented with 10% fetal calf serum (FCS), glutamine (5 mM) and penicillin/streptomycin (100 U/100 μg ml$^{-1}$). Cells were grown on glass dishes for at least 3 days. To initiate hypoxia, glass dishes, without lids, were put in a polypropylene box fitted with an inlet–outlet system and the box was purged with continuous flow (500 ml min$^{-1}$) of 95% nitrogen, 5% carbon dioxide (BOC) for various periods. This system renders cells radiobiologically hypoxic within 1 h (Stratford et al, 1980; Sutherland et al, 1982), corresponding to an oxygen tension less than 400 p.p.m. Duration of hypoxic exposure was defined from the start of nitrogen purging.

### Cell adhesion assay

Ninety-six-well plates, untreated for cell culture (Costar), were coated with collagen type I (10 μg ml$^{-1}$), fibronectin (10 μg ml$^{-1}$)

or vitronectin (5 μg ml$^{-1}$) either overnight at 4°C or 90 min at 37°C, washed twice with phosphate-buffered saline (PBS) and blocked in 0.1% bovine serum albumin (BSA) in PBS for 1–2 h at 37°C. The plates were re-washed with PBS just before addition of the cells. Cells (either exposed to hypoxia or left under normoxic conditions at 37°C) were trypsinized, washed once with full medium and twice in serum-free medium. Cells were resuspended in serum-free MEM at 1×10$^{6}$ cells ml$^{-1}$ and 50 μl were added to coated wells in quadruplicate. The same number of cells was also added to uncoated, but BSA-blocked, wells on the same plate to act as a negative adherence (blank) control. In some experiments, TS2/16 antibody (anti-β1; 18 μg ml$^{-1}$ final concentration) was added to the cells. Plates were then incubated at 37°C for 30–45 min. Unbound cells were washed off gently in PBS by immersion and flicking; 100 μl of growth medium was then added to adherent cells and the plates left at 37°C for a further 30 min. At the same time, 50 μl aliquots from the same untreated and hypoxia-treated cells were added to a tissue culture 96-well plate in quadruplicate, to act as a positive input or as the total cell count to be used in the calculation of percentage cell adhesion. The adhesion assay was carried out in such a way to avoid using multiple control samples, by terminating the different hypoxic exposures at the same time and measuring adhesion of all hypoxic samples and the control (normoxic, 0 h hypoxia) on one plate at the same time.

Adherent cells and total cell counts were quantified by the CellTiter 96 AQueous Non-Radioactive Cell Proliferation Assay (Promega). This assay uses an MTT derivative (MTS), which is a tetrazolium salt, and an electron-coupling reagent, phenazine methosulphate (PMS). MTS is bioreduced to a formazan that is soluble in tissue culture medium. The absorbence of the formazan at 492 nm can be measured directly from 96-well plates. Freshly prepared reagent (20 μl) was added to cells in 100 μl of growth medium. Plates were incubated at 37°C for 1–2 h and read at 492 nm using a plate reader. We confirmed that this assay was linear with cell number.

Cell adhesion of each sample was calculated as follows:

% Adhesion = [Mean OD (adherent cells) – mean OD (blank adherent cells) ×100] / [Mean OD (total cells) –mean OD (blank total cells)]

Data are expressed as the percentage adhesion ± s.d. of % adhesion (calculated from the combination of the s.d. and the individual OD values, according to Squires, 1968). Statistical analysis of the significance of observed differences between samples and controls was carried out by comparisons of all pairs using Tukey–Kramer HSD in the SAS JMP statistics package ($P < 0.05$ was considered to be significant).

### Cell viability/colony-forming ability

The MTS assay above only measures metabolically active cells, thus it was used to determine viability and reproductive integrity of cells. After cells were either trypsinized or detached by hypoxic exposure, 5 × 10$^{4}$ cells were added in quadruplicate to a 96-well culture plate and assayed 1 h after plating to compare viability of hypoxic and untreated cells. Cell viability was also confirmed by trypan blue exclusion.

To determine the proliferative capacity of untreated and hypoxia-detached cells, 1 × 10$^{4}$ cells per well were added in quadruplicate to a 96-well cell culture plate and assayed with MTS after 3 days of growth. The plating efficiency (PE) of hypoxia-detached and untreated cells was determined by plating known numbers of cells in cell culture flasks and counting the number of colonies that arose after 10 days of growth.

**Figure 1** Effect of exposure to hypoxia on adhesion of HMB-2 cells to fibronectin substrate. Hypoxia-treated and normoxic cells (0 h hypoxia) were trypsinized, washed and adhesion to fibronectin was measured as described in Materials and methods. Cells detached by longer hypoxic exposures (no trypsinization) were washed in serum-free medium and assayed for adhesion to fibronectin (horizontal rectangle represents the area where initial cell detachment from growth vessel occurred, i.e. 4–5 h for confluent and 9–10 h for exponential cells). The experiment shown is representative of five independent experiments. *$P < 0.05$

### Flow cytometry

After detachment by trypsin treatment and recovery in full growth medium (30 min), cells were washed in ice-cold PBS containing 0.1% BSA and 0.1% sodium azide and resuspended at 10$^{6}$ cells ml$^{-1}$. Several monoclonal antibodies were used in this study (final concentrations in brackets): L230 (anti-αv, 18 μg ml$^{-1}$) and TS2/16 (anti-β1, 12 μg ml$^{-1}$) were obtained from the American Type Tissue Culture Collection. LM609 (anti-αvβ3, 1:100; Chemicon International), P4H9 (anti-β2, 1:100; Life Technologies), p2A1 (anti-CD44, diluted hybridoma supernatant; Marshall et al, unpublished), ERIC-1 (anti-NCAM, 18 μg ml$^{-1}$; a gift from Dr J Kemshead, Bristol) were also used. Control antibody was non-immune mouse IgG (Dako). Cells were incubated with MAbs at 4°C for 30–45 min. The cells were then washed three

© Cancer Research Campaign 1998

**Figure 2** Effect of hypoxia (4 h) on adhesion of HMB-2 cells to collagen in absence or presence of anti-integrin antibodies. The anti-β1 antibody (TS2/16; 12 μg ml⁻¹ final concentration) induced adhesion of HMB-2 cells to collagen. The experiment is one of three similar independent experiments. *$P < 0.05$

times. Secondary FITC-anti-mouse IgG (Sigma) was added (1:120 dilution) to cells for 30 min at 4°C, then washed three times and resuspended in 0.5 ml of PBS. Finally, cells were analysed for surface integrin expression by flow cytometry (FACScan, Becton Dickinson) using the Lysis II program.

### RESULTS

### Cell adhesion

The adhesion of HMB-2, DX3 and HT29 cells to extracellular matrix proteins (collagen, fibronectin and vitronectin) was tested in initial experiments and it was found that HMB-2 cells adhere mainly to fibronectin and vitronectin, with very little adherence to collagen. DX3 cells showed substantial levels of adherence to all three substrates, while HT29 cells mainly adhered to collagen with less, but still significant, levels of binding to fibronectin and vitronectin.

Figure 1 shows a representative experiment in which exposure of HMB cells to hypoxia caused a reduction in cell adhesion to fibronectin. The extent and rate of loss of adhesion was highly dependent on the growth phase of the cell culture. Confluent cells lost their adhesive capacity much faster than exponentially growing cells; preliminary data (not shown), using conditioned medium of confluent cells on exponentially growing cells, show that the speed of loss of adhesion is dependent on both cell density and factors in the culture medium. DX3 and HT29 cells behaved in a similar way to HMB cells and adhesion to all extracellular matrices tested was reduced in a similar fashion (data not shown).

Adherence of HMB-2 cells to collagen, which was already very low, was apparently reduced even further by hypoxia. However, the use of anti-integrin activating antibodies, such as TS2-16 (anti-β1), showed that, in both control and hypoxia-exposed cells, adhesion was immediately and significantly increased by treatment with this functional activator, as shown in Figure 2. The level of adhesion of both HMB-2 cells (Figure 3) and DX3 cells (data not shown) returned to normal levels within 24 h of reoxygenation, with the rate of return depending on the extent of hypoxic exposure and growth phase.

**Figure 3** Effect of reoxygenation on cell adhesion. Confluent HMB-2 cells (**A**) were exposed either to 2 h of hypoxia (to cause a medium loss of adhesion) or to 5 h of hypoxia (to cause cell detachment) then allowed to recover in an incubator gassed with 5% carbon dioxide in air. Exponential HMB-2 cells (**B**) behaved in a similar manner but the hypoxic exposure had to be longer (either 5 or 9 h) to achieve similar time scales. *$P < 0.05$

### Cell detachment

Hypoxia caused eventual detachment of cells from the growth vessels. Continuous observation of cells under hypoxia showed that the detachment process is rapid and complete within 30 min after detachment is initiated. Cells detached in confluent layers or

© Cancer Research Campaign 1998

**Figure 4** Effect of hypoxia/reoxygenation on cell viability/growth. HMB-2 cells were detached completely by hypoxia (4 h) and plated in 96-well plates at similar cell numbers to untreated, trypsinized cells. Cells were assayed after 1 h (viability) or 3 days (growth). At the same time, cells were plated at known numbers in cell culture flasks to be assayed for colony formation 10 days later small multicellular aggregates termed 'flakes' (80% flakes, 20% single cells are estimated), hence it was possible to observe the onset of detachment by the naked eye. The suggestion from these empirical observations is that adhesion to the extracellular matrix is lost before the loss of cell–cell contact. Aspiration of these flakes by gentle pipetting resulted in the formation of a single-cell suspension. Hypoxia-detached cells retained about 60% of adhesion compared with untreated cells (the horizontal box in Figure 1 shows the area where initial cell detachment is observed). Confluent cells detached sooner (5–6 h) than exponentially growing cells (10 h). Further hypoxia treatment of detaching cells resulted in further loss of adhesion to extracellular components (Fibronectin), as shown in Figure 1, and caused cell–cell detachment and the formation of a single-cell suspension. Therefore, the initial detachment of cells was used as a marker point to terminate the hypoxic exposure of cells so as to retain cell viability.

### Cell viability/colony-forming ability

Exposure of HMB and DX3 cells to hypoxia sufficient to detach cells had no effect on cell viability and the capacity of cells to grow and form new colonies. This was most evident when hypoxia-detached cells were found to be able to reattach within a couple of hours, in a similar way to trypsinized untreated cells, and to continue growth after reoxygenation if left in the original growth vessel or when transferred to new culture vessels. Figure 4 shows data comparing the viability of untreated and hypoxia-detached cells using the MTS cell proliferation assay and the colony-forming assay. The data show that detachment of cells by hypoxia had no effect on cell viability or colony-forming ability of these cells. Plating efficiency (PE) of hypoxia-detached cells seemed to be slightly higher.

### Flow cytometry: integrin expression

Flow cytometric analysis revealed that exposure to hypoxia was accompanied by marked down-regulation of integrin expression, thus suggesting a possible mechanism for the observed reduction in adhesion. Hypoxia caused a marked down-regulation of β1, αv, αvβ3 (around 40% in detaching cells), while no change was observed with the negative control β2 integrin (Figure 5). To determine whether the down-regulation of cell adhesion molecules upon hypoxic exposure of cells was specific to integrins, we investigated two other cell adhesion molecules that are expressed on HMB-2 and DX3 cells, CD44 and N-CAM. It was found that hypoxia treatment also down-regulated these two adhesion molecules (Figure 5). Integrin expression returned to normal levels within 24 h after reoxygenation; the kinetics of β1 integrin down-regulation and recovery are shown in Figure 6.

### DISCUSSION

The effects of exposure to hypoxia on adhesion of cells to extracellular matrices and on changes in selected integrin expression in human tumour cell lines were investigated. Modulation of integrin activity by alterations in the conformation of the alpha and beta subunits as well as increased or decreased integrin expression can all lead to changes in cell adhesion. Most relevant studies in the literature have concentrated on studying the effect of hypoxia on adhesion of normal cells, such as haemopoietic and endothelial cells, with no information on neoplastic cells. It was shown that hypoxia increased adhesion of leucocytes and polymorphonuclear cells to endothelial cells (Milhoan et al, 1992; Arnould et al, 1993, 1995; Ginis et al, 1995; Klein et al, 1995) and that this effect was attributable to increased expression of endothelial cell adhesion molecules. In another investigation, hypoxia was shown to decrease adhesion of granulocytes to endothelial cells (Pietersma et al, 1994), suggesting that adhesive responses to this stimulus might represent a cell lineage-specific response.

In the present study, hypoxia caused down-regulation of cell adhesion molecules, including integrins, at the surface of human tumour cells, and this was linked to the reduction of adhesion to extracellular matrix components, which in turn led to cell detachment. The extent and rate of loss and recovery of adhesion/integrin expression was dependent on the growth phase of the cells. The reason for the variation in the response of exponential and confluent cells to hypoxic stress is not known and is currently under investigation. Preliminary data indicate that it is dependent

 © Cancer Research Campaign 1998

**Figure 5** Effect of hypoxia on the expression of cell surface adhesion molecules. Confluent HMB-2 cells were detached by hypoxia (5 h) and labelled with different antibodies against cell-surface adhesion molecules. All expressed integrins assayed were reduced (50–70% of mean value), whereas there was no change in the β2 integrins included as negative control. The data shown were obtained from one of three similar, independent experiments on both cell density and factors in the medium. Differences in the capacity of exponential and confluent cells to adhere to extracellular matrices is also under investigation.

The finding that hypoxic cells can lose and regain attachment to extracellular matrices accompanied by loss and recovery of integrin expression has implications for the potential behaviour of hypoxic cells in solid tumours in vivo. Cells that detach from hypoxic regions of tumours could have the opportunity to migrate to distant sites in normal tissue to provide foci for new tumour growth. It has been shown recently that relatively small changes in integrin expression or affinity can lead to substantial changes in migration spread (Palacek et al, 1997). This suggests that hypoxic conditions could have a significant impact on the invasive and migratory behaviour of malignant cells.

It is interesting to note (Figure 2) that the effects of loss of β1 integrin expression could be offset by the application of the

© Cancer Research Campaign 1998

**Figure 6** Kinetics of reduction/recovery in integrin expression after hypoxia/reoxygenation of HMB cells. This figure shows the reduction and recovery after 4 h (confluent) or 6 h (exponential) of hypoxia of β1 integrin subunit. This is one of three independent experiments

β1-activating antibody TS2/16. It is too early to say whether such an application could be used to prevent or attenuate loss of adhesion in case this was to be found therapeutically beneficial. Thus, at this time, we cannot rule out the possibility that the hypoxia-induced down-regulation of integrin expression is exacerbated by inactivation of integrin function.

Our data are consistent with the findings of Young et al (1988) and Young and Hill (1990), who reported that successive treatment of mouse tumour cells (KHT and B16) in vitro with hypoxia and reoxygenation greatly enhanced their ability to form lung tumours in recipient mice. However, in these studies, the authors reported no such hypoxic detachment of viable cells, but it was reported that prolonged exposure to extreme hypoxia resulted in loss of cell viability and caused detachment of cells, which were found to be non-proliferating. The detachment of viable cells is an interesting finding as it is commonly perceived that detachment of cells by stress-inducing agents is indicative of cell death by apoptosis. Hypoxia is known to be toxic to cells in culture (Shrieve et al, 1983; Spiro et al, 1984) and is also reported to induce DNA damage-independent apoptosis in cells, including those of the HT29 line (Yao et al, 1995). This might help to explain our finding that HT29 only partly recovered after detachment by hypoxia. However, this is not a straightforward issue, as Graeber et al (1996) have reported that p53 mutant cells are more resistant than cells with a p53 wild-type phenotype to hypoxia-induced apoptosis; this therefore seems contrary to our observations as HT29 is known to be mutant (e.g. Lawrence et al, 1996). Both HMB-2 and DX3 have been reported as wild-type p53 (Montano et al, 1994), however our own assessment of these cell lines using the FASAY method (Lomax et al, 1997) indicates that HMB-2 expresses mutant p53 (JF Marshall, personal communication).

In summary, we have shown that hypoxia reduces adhesion to extracellular matrix molecules and down-regulates integrin expression, among other cell adhesion molecules, in human melanoma cells. The hypoxic response appears not to be specific for a particular extracellular substrate or integrin but represents a general phenomenon. Hypoxia appears to induce detachment of cells via these mechanisms and the hypoxia-detached cells are viable and will form new foci of growth if reoxygenated.

### REFERENCES

Arnould T, Michiels S and Remacle J (1993) Increased PMN adherence on endothelial cells after hypoxia. *Am J Physiol* **264**: C1102–1110

Arnould T, Michiels S, Janssens D, Delaire E and Remacle J (1995) Hypoxia induces PMN adhesion to endothelium. *Cardiovasc Res* **30**: 1009–1016

Brizel DM, Scully SP, Harrelson JM, Prosnitz LR and Dewhirst MW (1996) Tumor oxygenation predicts for the likelihood of distant metastases in human soft tissue sarcoma. *Cancer Res* **56**: 941–943

Ginis I, Mentzer SJ, Li X and Faller DV (1995) Characterization of a hypoxic-responsive adhesion molecule for leucocytes on human endothelial cells. *J Immunol* **155**: 802–810

Graeber TG, Osmanian C, Jacks T and Giaccia AJ (1996) Hypoxia-mediated selection of cells with diminished apoptotic potential in solid tumours. *Nature* **379**: 88–91

Hart IR (1996) Adhesion receptors and cancer. In *Molecular Biology of Cell Adhesion Molecules*, Horton MA. (ed.), pp 87–98

Hasan NM, Parker PJ and Adams GE (1996) Induction and phosphorylation of PKC by hypoxia and radiation. *Radiat Res* **145**: 128–133

Hockel M, Schlenger K, Aral B, Mitze M, Schaffer U and Vaupel P (1996) Association between tumor hypoxia and malignant progression in advanced cancer of the uterine cervix. *Cancer Res* **56**: 4509–4515

Hynes RO (1992) Integrins: versatility, modulation and signalling in cell adhesion. *Cell* **69**: 11–25

Juliano RL (1987) Membrane receptors for extracellular matrix macromolecules: Relationship to cell adhesion and tumor metastasis. *Biochim Biophys Acta Rev Cancer* **907**: 261–278

Juliano RL and Varner JA (1993) Adhesion molecules in cancer: the role of integrins. *Curr Opin Cell Biol* **5**: 812–818

Klein CL, Kohler H, Bittinger F and Kirkpatrick CJ (1995) Comparative studies on vascular endothelium in vitro. Hypoxia and cell adhesion. *Pathobiology* **63**: 1–8

Lawrence TS, Davis MA and Loney TL (1996) Fluoropyrimidine-mediated radiosensitization depends on cyclin E-dependent kinase activation. *Cancer Res* **56**: 3203–3206

Lomax ME, Barnes DM, Gilchrist R, Picksley SM, Varley JM and Camplejohn RS (1997) Two functional assays employed to detect an unusual mutation in the oligomerisation domain of *p53* in a Li–Fraumeni like family. *Oncogene* **14**: 1869–1874

Milhoan KA, Lane TA and Bloor CM (1992) Hypoxia induces endothelial cells to increase their adherence for neutrophils. *Am J Physiol* **263**: H956–H962

Montano X, Shamsher M, Whitehead P, Dawson K and Newton J (1994) Analysis of p53 in human cutaneous melanoma cell lines. *Oncogene* **9**: 1455–1459

Onoda JM, Piechocki MP and Honn KV (1992) Radiation induced expression of integrins in melanoma cells. *Radiat Res* **130**: 281–288

Palecek SP, Loftus JC, Ginsberg MH and Horwitz AF (1997) Integrin–ligand binding properties govern cell migration speed through cell–substratum adhesiveness. *Nature* **385**: 537–540

*© Cancer Research Campaign 1998*

Pietersma A, de-Jong N, Koster JF and Sluiter W (1994) Extreme hypoxia decreases the adherence of granulocytes to endothelial cells in vitro. *Ann NY Acad Sci* **723**: 486–487

Shrieve DC, Deen DF and Harris JW (1983) Effects of extreme hypoxia on the growth and viability of EMT6/SF mouse tumour cells in vitro. *Cancer Res* **43**: 3521–3527

Spiro IJ, Rice GC, Durand RE and Ling CC (1984) Cell killing, radiosensitization and cell cycle distribution induced by chronic hypoxia. *Int J Radiat Oncol Biol Phys* **10**: 1275–1280

Squires GL (1968) *Practical Physics*, p. 37. McGraw-Hill: New York.

Stratford IJ, Williamson C and Adams GE (1980) Combination studies with misonidazole and a cis-platinum complex. *Br J Cancer* **41**: 517–520

Sutherland RM, Keng P, Conroy PJ, McDermott D, Bareham BJ and Passalacqua W (1982) In vitro hypoxic cytotoxicity of nitroimidazoles: uptake and cell cycle phase specificity. *Int J Radiat Oncol Biol Phys* **8**: 745–748

Yao KS, Clayton M and O'Dwyer PJ (1995) Apoptosis in human adenocarcinoma HT29 cells induced by exposure to hypoxia. *J Natl Cancer Inst* **87**: 117–121

Young SD and Hill RP (1990) Effects of reoxygenation on cells from hypoxic regions of solid tumors: anticancer drug sensitivity and metastatic potential. *J Natl Cancer Inst* **82**: 371–380

Young SD, Marshall RS and Hill RP (1988) Hypoxia induces DNA overreplication and enhances metastatic potential of tumor murine cells. *Proc Natl Acad Sci USA* **85**: 9533–9537

© Cancer Research Campaign 1998